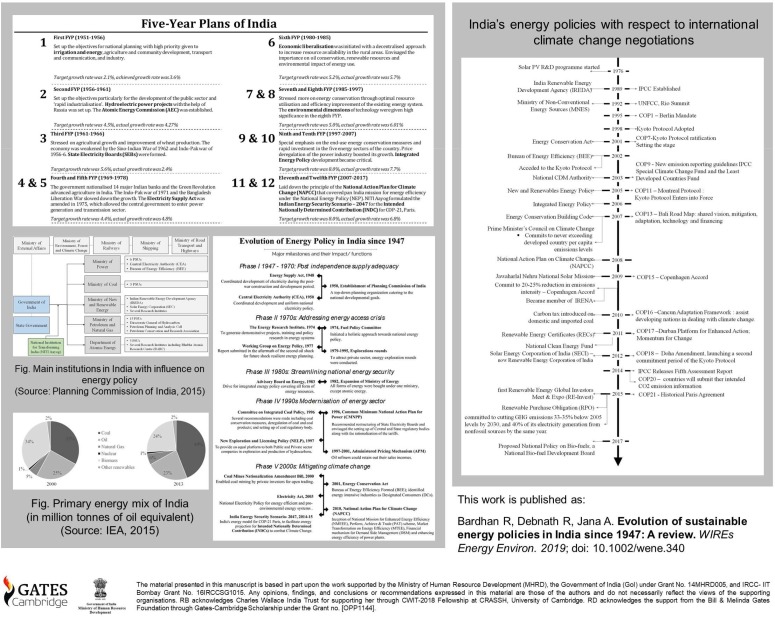# Evolution of sustainable energy policies in India since 1947: A review

**DOI:** 10.1002/wene.340

**Published:** 2019-08-15

**Authors:** Ronita Bardhan, Ramit Debnath, Arnab Jana

**Affiliations:** a Centre for Research in Arts, Social Sciences and Humanities, University of Cambridge, Cambridge CB3 9DT, United Kingdom; b Behaviour and Building Performance Group, Department of Architecture, University of Cambridge, CB2 IPX, United Kingdom; c Centre for Urban Science and Engineering, Indian Institute of Technology Bombay, Mumbai 400076, India

## Abstract

India's Intended Nationally Determined Contributions in 2015 toward the Two‐Degree Celsius climate change goal has endorsed 15% of renewable integration in the primary energy mix by 2020. The energy space is strategy to meet the target without affecting its immediate sustainable development goals. This study documents this strategic effort by tracking the historical trajectory of energy policy planning since its independence in 1947. An objective ontological approach was adopted in reviewing the evolution of energy policy into five distinct phases. Phase I (1947–1970), focused on supply adequacy with the overall thrust on infrastructure development as the pillar of Indian economy. In Phase II (the 1970s) the focus shifted in addressing the energy access crisis. Phase III (the 1980s) was based on increment, diversification, and streamlining on supplies for energy security purposes. Phase IV (the 1990s) is the period of modernization of the overall Indian electricity system. Phase V (the 2000s) is the present phase of market transformation and climate change mitigation energy policies. A co‐assessment of India's policy to the international climate negotiations showed that India remained responsive to international climate goals. It became reactive in the planning for sustainable energy policy after its ratification of Kyoto Protocol in 2001. Since then, India has been instrumental in administering strict emission reduction norms and efficiency measures. This review concludes that the country needs to upgrade its inefficient transmission and distribution networks, which was broadly neglected. The subsidy allocations in domestic energy resources should be well‐adjusted without compromising on its social costs.

**Fig:**
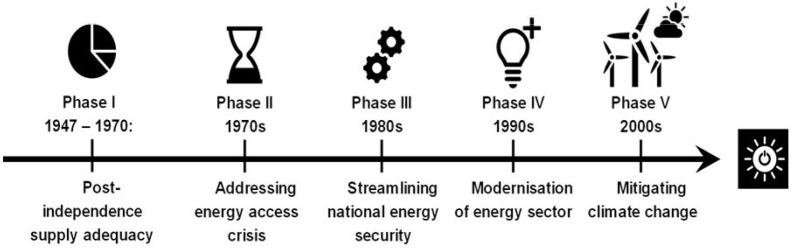
Chronology of energy policy phases in india since 1947

**Figure:**